# Substance P—Friend or Foe

**DOI:** 10.3390/jcm11133609

**Published:** 2022-06-22

**Authors:** Prema Robinson, Emma Rodriguez, Miguel Muñoz

**Affiliations:** 1 Department of Infectious Diseases, Infection Control and Employee Health, The University of Texas MD Anderson Cancer Center, Houston, TX 77030, USA; eerodriguez1@mdanderson.org; 2 Research Laboratory on Neuropeptides (IBIS), Pediatric Intensive Care Unit, Virgen del Rocío University Hospital, Avda. Manuel Siurot s/n, 41012 Sevilla, Spain

Substance P (SP), a neuropeptide and pain transmitter has multiple roles and is involved in various processes in the body. Dating back to 1931, SP was isolated by Euler and Gaddum as a crude extract from equine brain and intestine [1]. SP undecapeptide was sequenced by Leeman’s lab [2]. The main functional role of SP includes pain transmission/perception, enhancement of survival, immunostimulatory functions and induction of chemotaxis of immune cells, immune responses to few sparse microbes, and modulation of cardiovascular processes, outlined as follows:

(1) SP is involved in transmitting pain impulses [3]. However, although SP is one of the predominant neuropeptide that is involved in transmitting pain impulses, there are several other neuropeptides that transmit pain impulses, such as vasoactive intestinal peptide [4], calcitonin gene-related peptide [5], and cholecystokinin [6].
(2) SP has various effects on immune cells [7] as follows: SP enhances the survival and immunostimulatory capacity of dendritic cells [8,9,10,11,12]. SP enhances IL-12 production in macrophages [7,13]. SP inhibits spontaneous apoptosis while causing chemotaxis of eosinophils [7,14,15]. SP induces superoxide production, phagocytosis, and chemotaxis of neutrophils [7,16,17,18]. SP induces mast cell degranulation and modulates NK cell cytotoxicity [7]. SP promotes proliferation of activated T-cells and induces generation of memory TH17 cells [19,20,21]. However, SP is not indispensable; there are several other mediators that induce the above effects on immune cells.
(3) There are few and sparse reports that SP plays a beneficial role in microbial infections, for example the absence of SP leads to the host immunity to murine gamma herpes virus 68 (HV-68) and genital herpes virus (HSV-2) infection being significantly compromised [22,23]. In addition, SP has been shown to augment immunity to Salmonella in a murine model [24]. However, there are contrasting derogatory roles of SP in other important microbial infections that is outlined below.
(4) SP has shown to be protective following acute ischemia-reperfusion. For example, in acute ischemia reperfusion studies, SP has been shown to provide important vasodilatory effects that presumably appear to be protective initially by increasing myocardial reperfusion. However, there are contrasting derogatory roles of SP in the cardiovascular setting that is outlined below.

## 1. Derogatory Role of SP

SP signaling induces emesis. High levels of SP are found in postrema and the nucleus solitarius in the central nervous system (CNS), two areas that control the vomiting reflex. Cisplatin and other systemic chemotherapies cause the release of emetogenic SP. Blockage of SP signaling lessens the severity of chemotherapy-induced emesis [25]. Most importantly, SP is involved in tumor growth and development of both solid and non-solid cancers, SP induces mitogenesis, migration (leading to invasion and metastasis), anti-apoptosis (or survival) of tumor cells [26,27,28,29,30]. Although SP has shown to be beneficial in HV-68, HSV-2, and Salmonella infection there are other important microbial infections wherein SP has been shown to be harmful. For example, SP has been shown to enhance the replication of HIV in blood-isolated mononuclear phagocytes [31,32]. SP receptor antagonist, aprepitant, has been shown to reduce viral load in SIV-infected macaques, and reduce the pro-inflammatory cytokines in HIV positive individuals, thus implying that SP receptor antagonism may have the possibility for consideration as an adjunct therapy in HIV infection [33]. In addition, SP receptor antagonist has shown to limit Sendai virus-induced bronchoconstriction in guinea pigs [34]. Furthermore, targeting of SP receptor was shown to limit neuroinflammation in a murine model of pneumococcal meningitis [35]. Finally, mice deficient in SP receptor, have demonstrated a decreased level of inflammatory cytokines after CNS infection of two clinically relevant bacterial CNS pathogens—Neisseria meningitides and Borrelia burgdorferi [36]. In the cardiovascular setting, long-term up-regulation of substance P appears to induce detrimental responses in the form of inflammation, apoptosis, MMP activation, and changes to the extracellular matrix, as observed in myocarditis, volume overload, and magnesium-deficiency. Additionally, SP contributes to the cardiotoxicity induced by chemotherapeutic agent; doxororubicin [37]. SP signaling has shown to contribute to the pathogenesis of several skin conditions, such as eczema, dermatitis, psoriasis, and rosacea [38,39]. SP is implicated in the pathogenesis of pulmonary conditions/diseases, such as asthma and chronic bronchitis. SP induces constriction of bronchial smooth muscle cells, which reduces the airway diameter and triggers mast cell degranulation in lung tissue [3]. SP signaling has shown to contribute to the pathogenesis of several inflammatory conditions/diseases, such as inflammatory bowel disease, pancreatitis, epilepsy and seizure disorders, cryptosporidiosis, myocarditis, and rheumatoid arthritis [40,41,42,43,44,45,46].

Lastly, SP has been shown to be involved in the pathogenesis of diseases including, but not limited to, diabetes, heart failure, and migraine [39].

## 2. Conclusions

SP is a paradigmatic peptide that can be considered as friend or foe. However, the derogatory effects of high concentrations of SP far outweighs its beneficial effects.

## Data Availability

Not applicable.
